# IMMUNO-COV v2.0: Development and Validation of a High-Throughput Clinical Assay for Measuring SARS-CoV-2-Neutralizing Antibody Titers

**DOI:** 10.1128/mSphere.00170-21

**Published:** 2021-06-02

**Authors:** Rianna Vandergaast, Timothy Carey, Samantha Reiter, Chase Lathrum, Patrycja Lech, Clement Gnanadurai, Michelle Haselton, Jason Buehler, Riya Narjari, Luke Schnebeck, Anne Roesler, Kara Sevola, Lukkana Suksanpaisan, Alice Bexon, Shruthi Naik, Bethany Brunton, Scott C. Weaver, Grace Rafael, Sheryl Tran, Alina Baum, Christos A. Kyratsous, Kah Whye Peng, Stephen J. Russell

**Affiliations:** a Imanis Life Sciences, Rochester, Minnesota, USA; b Vyriad, Inc., Rochester, Minnesota, USA; c Mayo Clinic Department of Molecular Medicine, Rochester, Minnesota, USA; d World Reference Center for Emerging Viruses and Arboviruses, University of Texas Medical Branch, Galveston, Texas, USA; e Institute for Human Infections and Immunity, University of Texas Medical Branch, Galveston, Texas, USA; f Department of Microbiology and Immunology, University of Texas Medical Branch, Galveston, Texas, USA; g Regeneron Pharmaceuticals Inc., Tarrytown, New York, USA; University of Saskatchewan

**Keywords:** COVID-19, SARS-CoV-2, antibody titer, clinical validation, high-throughput assay, neutralizing antibodies, surrogate virus

## Abstract

Neutralizing antibodies are key determinants of protection from future infection, yet well-validated high-throughput assays for measuring titers of SARS-CoV-2-neutralizing antibodies are not generally available. Here, we describe the development and validation of IMMUNO-COV v2.0, a scalable surrogate virus assay, which titrates antibodies that block infection of Vero-ACE2 cells by a luciferase-encoding vesicular stomatitis virus displaying SARS-CoV-2 spike glycoproteins (VSV-SARS2-Fluc). Antibody titers, calculated using a standard curve consisting of stepped concentrations of SARS-CoV-2 spike monoclonal antibody, correlated closely (*P* < 0.0001) with titers obtained from a gold standard 50% plaque-reduction neutralization test (PRNT50%) performed using a clinical isolate of SARS-CoV-2. IMMUNO-COV v2.0 was comprehensively validated using data acquired from 242 assay runs performed over 7 days by five analysts, utilizing two separate virus lots, and 176 blood samples. Assay performance was acceptable for clinical use in human serum and plasma based on parameters including linearity, dynamic range, limit of blank and limit of detection, dilutional linearity and parallelism, precision, clinical agreement, matrix equivalence, clinical specificity and sensitivity, and robustness. Sufficient VSV-SARS2-Fluc virus reagent has been banked to test 5 million clinical samples. Notably, a significant drop in IMMUNO-COV v2.0 neutralizing antibody titers was observed over a 6-month period in people recovered from SARS-CoV-2 infection. Together, our results demonstrate the feasibility and utility of IMMUNO-COV v2.0 for measuring SARS-CoV-2-neutralizing antibodies in vaccinated individuals and those recovering from natural infections. Such monitoring can be used to better understand what levels of neutralizing antibodies are required for protection from SARS-CoV-2 and what booster dosing schedules are needed to sustain vaccine-induced immunity.

**IMPORTANCE** Since its emergence at the end of 2019, SARS-CoV-2, the causative agent of COVID-19, has caused over 100 million infections and 2.4 million deaths worldwide. Recently, countries have begun administering approved COVID-19 vaccines, which elicit strong immune responses and prevent disease in most vaccinated individuals. A key component of the protective immune response is the production of neutralizing antibodies capable of preventing future SARS-CoV-2 infection. Yet, fundamental questions remain regarding the longevity of neutralizing antibody responses following infection or vaccination and the level of neutralizing antibodies required to confer protection. Our work is significant as it describes the development and validation of a scalable clinical assay that measures SARS-CoV-2-neutraling antibody titers. We have critical virus reagent to test over 5 million samples, making our assay well suited for widespread monitoring of SARS-CoV-2-neutralizing antibodies, which can in turn be used to inform vaccine dosing schedules and answer fundamental questions regarding SARS-CoV-2 immunity.

## INTRODUCTION

On 11 March 2020, the World Health Organization declared COVID-19, caused by SARS-CoV-2, a pandemic. Since then, the coordinated efforts of numerous researchers, biotechnology and pharmaceutical companies, contract manufacturers, health care organizations, and governmental agencies have resulted in the approval and initial distribution of the first SARS-CoV-2 vaccines. Clinical trial data indicate that the vaccines currently approved in the United States are approximately 95% effective at preventing COVID-19 (1, 2). However, the durability of this protection is unknown. Neutralizing antibody responses following vaccination correlate with protective immunity (3–6), yet an increasing number of studies, including this one, demonstrate that neutralizing antibody levels fall steadily in the months following natural SARS-CoV-2 infection or vaccination (7–11). Thus, protective antibody responses, including those elicited by vaccination, may be relatively short lived, and repeat vaccine dosing over several years may be necessary to achieve and maintain herd immunity. It is not currently known what titer of neutralizing antibodies confers protection from SARS-CoV-2 infection or COVID-19. Studies to monitor neutralizing antibody responses and the associated risk of infection at various time points postvaccination are needed to inform decisions on the appropriate timing of booster vaccine doses. To facilitate these studies, a reliable, high-throughput method for quantitatively measuring neutralizing antibody titers is critically needed.

Over the course of the past year, numerous serological tests have been developed, and many have received Emergency Use Authorization (EUA) approvals for the detection of antibodies against SARS-CoV-2. These tests, which include enzyme-linked immunosorbent assays (ELISAs), high-throughput tests run in CLIA laboratories, and lateral flow-based point-of-care tests, provide a useful and convenient way to identify individuals previously infected with SARS-CoV-2. However, it is well known that only a small subset of virus-specific antibodies are capable of neutralizing virus infectivity and thereby protecting against future viral infection and disease (12). Importantly, the serological assays for which EUA approvals have been granted are not able to discriminate between neutralizing and nonneutralizing antibodies. Available evidence also suggests that postvaccination and postinfection neutralizing antibody titers do not correlate strongly with total antibody titers (10, 13–16), and it is unknown whether neutralizing antibody titers decay over time more rapidly than those of nonneutralizing antibodies. Thus, for reliable assessment of the level of protection against SARS-CoV-2 infection in vaccinated or previously infected individuals, neutralizing antibody assays are preferred.

The gold standard assay for the quantitation of virus neutralizing antibodies is the plaque-reduction neutralization test (PRNT). While providing a reasonable measure of the blood concentration of antibodies capable of neutralizing the SARS-CoV-2 virus, PRNT is labor-intensive and requires use of a clinical virus isolate, such that the test can be performed only under biosafety level 3 (BSL-3) containment. Safer alternative neutralization assays have been developed using nonreplicating lentiviral vectors (10, 14, 17, 18) or vesicular stomatitis viruses (VSVs) (19) pseudotyped with the SARS-CoV-2 spike glycoprotein. However, due to technical factors impacting the manufacture of these pseudotyped viruses, they are generally produced in small batches of variable titer, which significantly limits the scalability of these assays. The use of fully replication-competent VSVs expressing the SARS-CoV-2 spike protein provides an attractive alternative for the development of neutralizing assays (20–22), as they can be propagated extensively to generate much larger reagent stocks. Moreover, because the natural VSV glycoprotein (G) is replaced with the SARS-CoV-2 spike protein, these recombinant viruses mimic SARS-CoV-2 entry, which is initiated by binding of the spike protein to its receptor angiotensin-converting enzyme 2 (ACE2) on the cell surface (23–25). Once bound to ACE2 via its receptor binding domain (RBD), the spike protein is proteolytically cleaved by the cell surface transmembrane serine protease TMPRSS2 or by endosomal cysteine proteases cathepsin B/L, providing a critical trigger for subsequent membrane fusion and virus entry into the cell (23, 26). Studies have mapped the targets of SARS-CoV-2-neutralizing antibodies to diverse epitopes within the spike protein, and antibodies that block ACE2 receptor binding, spike protein cleavage, or subsequent conformational rearrangements of the spike protein that lead to membrane fusion are all strongly neutralizing (27–31).

**FIG 1:**
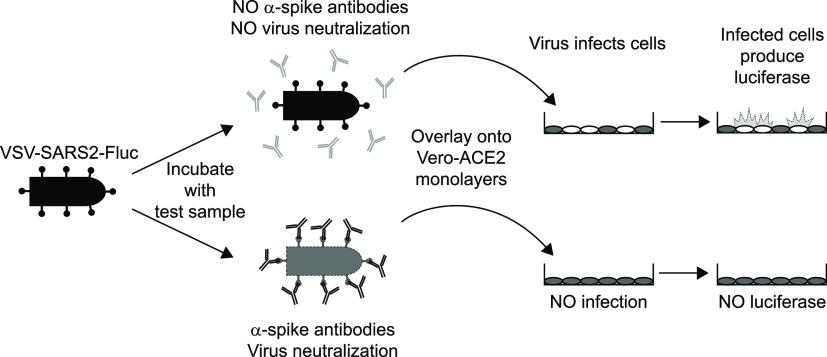
Overview of the IMMUNO-COV v2.0 assay. A VSV expressing SARS-CoV-2 spike and firefly luciferase (VSV-SARS2-Fluc) is incubated with test sera or plasma. In the absence of SARS-CoV-2-neutralizing antibodies (top), the virus retains infectivity and infects Vero-ACE2 monolayers. If the test sample contains SARS-CoV-2-neutralizing antibodies (bottom), the antibodies inhibit infection by blocking cell entry. As virus replication proceeds, infected cells express luciferase, which is used to quantitate virus infection. High luciferase signal means the test sample did not neutralize the virus, while decreased luciferase indicates the presence of SARS-CoV-2-neutralizing antibodies.

Here, we describe the development, optimization, and validation of IMMUNO-COV v2.0, a fully scalable neutralization assay that uses a replication-competent G cistron-deleted recombinant VSV encoding both the SARS-CoV-2 spike protein and firefly luciferase (Fluc) (Fig. 1). Over 23,000 vials of this virus were prepared and cryopreserved from a single large-production run, providing sufficient material to assay more than 5 million serum or plasma samples. Anti-SARS-CoV-2-neutralizing antibody titers determined using IMMUNO-COV v2.0 demonstrated strong linear correlation with titers obtained using the classical PRNT under BSL-3. IMMUNO-COV v2.0 assay performance has remained robust and accurate for at least 3 months, during which time we have conducted extensive validation testing and subsequent verification studies. In keeping with the observations of other groups (7, 8, 16, 28, 32), higher titers of neutralizing antibodies were observed in subjects recovering from more severe SARS-CoV-2 infections, though strong responses were also seen in several subjects who had only mild disease symptoms. Importantly, a substantial decline in neutralizing antibody levels was observed in most COVID-19 convalescent subjects who were tested repeatedly over a 6-month period, regardless of the initial antibody titer. Taken together, our results underscore the importance of monitoring neutralizing antibody titers over time and demonstrate how IMMUNO-COV v2.0 can be used to accurately quantify these responses at scale.

## RESULTS

### Generation of VSV-SARS2-Fluc.

**FIG 2:**
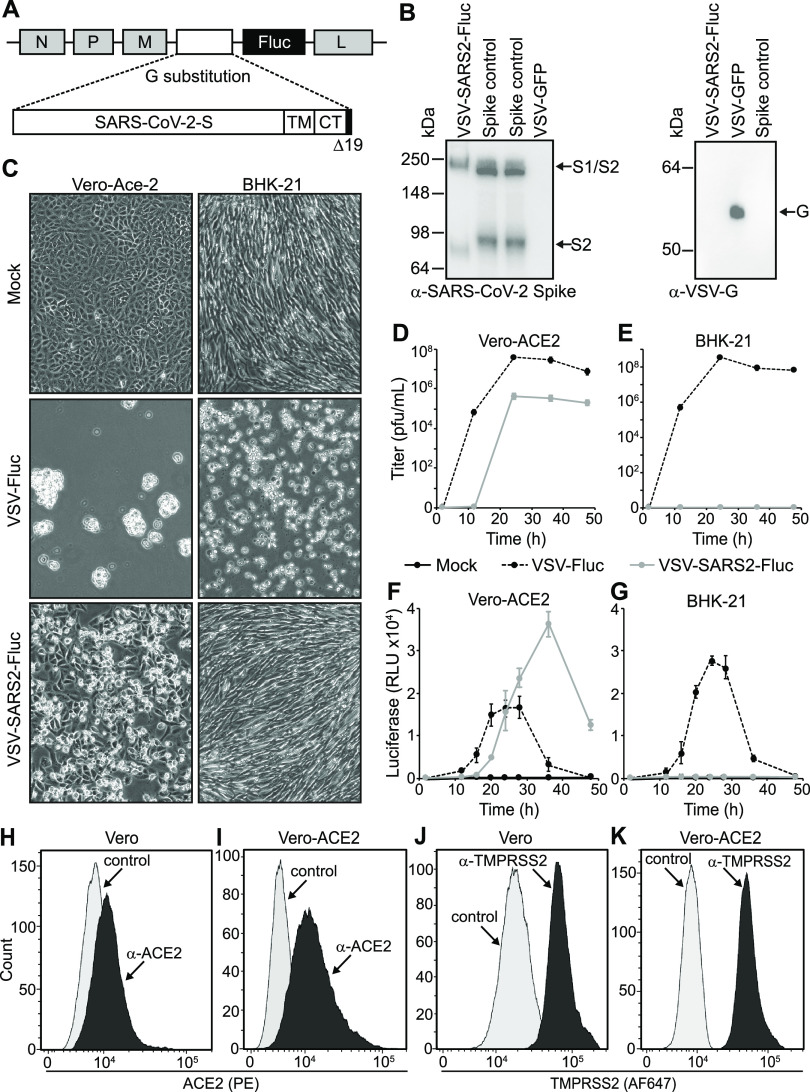
Generation and characterization of VSV-SARS2-Fluc. (A) Schematic representation of the VSV-SARS2-Fluc genome. The locations of the VSV N, P, M (M51R), and L genes are shown. In place of VSV-G, a codon-optimized SARS-CoV-2 spike gene with a 19-amino-acid C-terminal (CT) deletion (Δ19CT) is substituted. TM is the transmembrane domain. Firefly luciferase (Fluc) is inserted as an additional transcriptional element between S-Δ19CT and L. Not drawn to scale. (B) Immunoblot analysis. VSV-SARS2-Fluc or VSV-GFP control virus (5 × 10^5^ total PFU) or spike control from lysates of cells overexpressing SARS-CoV-2 spike was subjected to immunoblot analysis using anti-SARS-CoV-2 spike antibody (left) and anti-VSV-G antiserum (right). Arrows indicate the full-length S1/S2 variant and cleaved S2 variant of spike and the VSV-G proteins. (C) Infection of cell monolayers. Vero-ACE2 or BHK-21 cell monolayers were infected with VSV-SARS2-Fluc or control VSV-Fluc or mock infected. Images were taken 48 h postinfection at ×100 magnification. (D and E) Replication curves. Vero-ACE2 or BHK-21 cell monolayers were infected as in panel C, and the virus titers from culture supernatants collected at the indicated times postinoculation were determined. (F and G) Luciferase activity. Vero-ACE2 or BHK-21 cells were infected with VSV-SARS2-Fluc or control VSV-Fluc or mock infected in 96-well plates, and at the indicated times luciferase activity was measured. (H to K) Flow cytometry. Expression of ACE2 (H and I) and TMPRSS2 (J and K) was measured in Vero and Vero-ACE2 cells by flow cytometry using anti-ACE2 or anti-TMPRSS2, respectively. Controls were secondary antibody only.

Our previously published SARS-CoV-2 neutralization assay relied upon virus-induced fusion of two dual split protein (DSP) reporter cell lines to generate a luciferase signal (21). To further improve assay throughput and eliminate the need for two cell lines, we generated a recombinant VSV (VSV-SARS2-Fluc) encoding SARS-CoV-2 spike-Δ19CT (S-Δ19CT) in place of VSV-G and firefly luciferase (Fluc) as an additional transcriptional unit located between the S-Δ19CT and VSV-L genes (Fig. 2A). Cells infected with VSV-SARS2-Fluc express the virus-encoded luciferase, which is used to measure the level of virus infection. Incorporation of SARS-CoV-2 spike protein into VSV-SARS2-Fluc virions was confirmed by immunoblotting (Fig. 2B). VSV-SARS2-Fluc infection and replication were also dependent on cellular ACE2 expression. Robust VSV-SARS2-Fluc replication and virus-induced cell death were observed in Vero-ACE2 cells, which overexpress the SARS-CoV-2 receptor ACE2 (Fig. 2C and D), but not in hamster BHK-21 cells (Fig. 2C and E), which do not express human ACE2. The control virus VSV-Fluc, which encodes VSV-G but not S-Δ19CT, efficiently infected and replicated in Vero-ACE2 and BHK-21 cells. Cellular luciferase activity specifically correlated with replication of the Fluc-expressing viruses (Fig. 2F and G), with loss of luciferase signal at later time points coinciding with the death of infected cultures. Together, these data confirmed functional VSV-G replacement with S-Δ19CT and efficient Fluc expression from the VSV-SARS2-Fluc virus.

### Vero-ACE2 cells are an optimal cell substrate for detecting virus neutralization.

**FIG 3:**
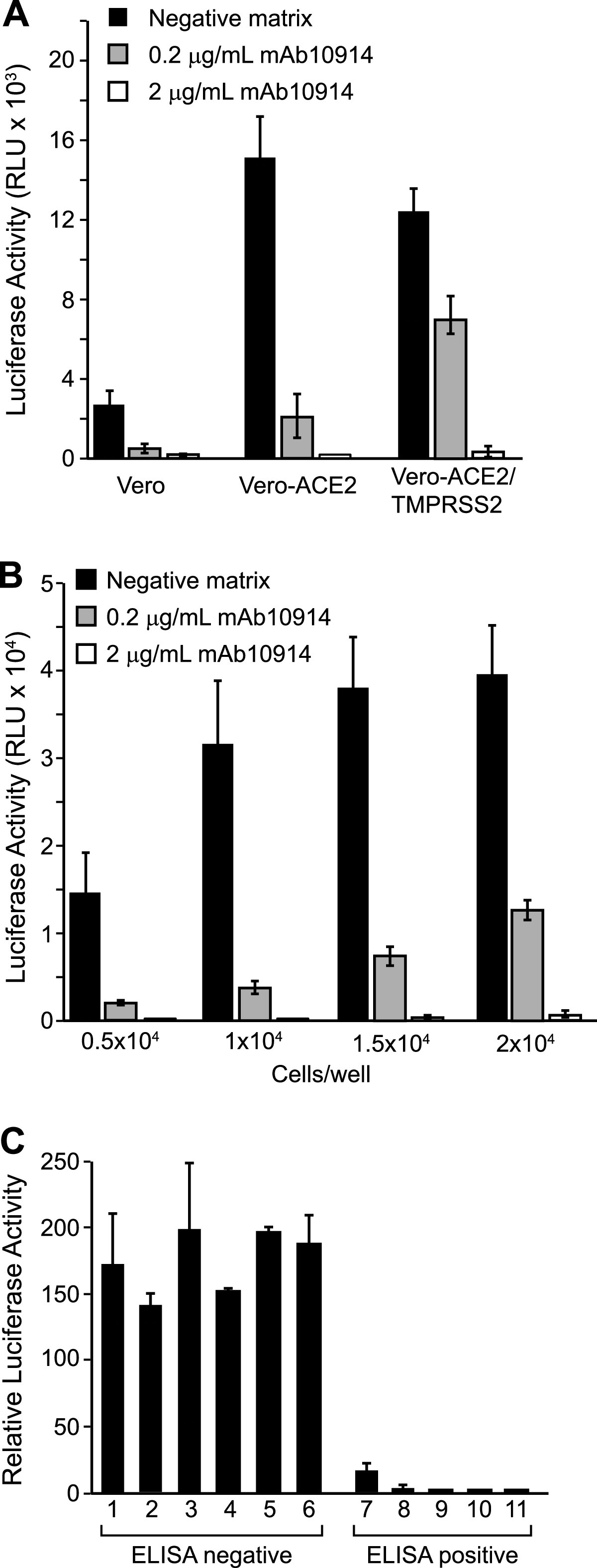
Inhibition of VSV-SARS2-Fluc by monoclonal antibodies and convalescent sera. (A) Infectivity of different Vero cell lines. VSV-SARS2-Fluc was incubated with 2 or 0.2 μg/ml of monoclonal anti-SARS-CoV-2 spike antibody mAb10914 in pooled seronegative sera or pooled seronegative sera alone (negative matrix). After 30 min, virus mixes were overlaid onto Vero, Vero-ACE2, or Vero-ACE2/TMPRSS2 cells. Luciferase activity was measured after an additional 24 h. Values represent the average (mean) relative light units (RLU) ± standard deviation. (B) Optimization of cell density. The indicated numbers of Vero-ACE2 cells were seeded in 96-well plates. The following day, virus mixes as described for panel A were overlaid onto the cell monolayers. Luciferase activity was measured after an additional 24 h. Values represent the average (mean) RLU ± standard deviation. (C) Neutralization by convalescent sera. VSV-SARS2-Fluc was incubated with pooled seronegative sera at a 1:80 dilution or serum samples from 11 donors (6 seronegative and 5 seropositive for anti-SARS-CoV-2 antibodies by ELISA) at a 1:80 dilution. After 30 min, virus-serum mixes were overlaid onto Vero-ACE2 cells. Luciferase activity was measured after an additional 24 h. Values represent average (mean) luciferase activity relative to the pooled seronegative serum sample control ± standard deviation.

VSV-SARS2-Fluc infects Vero cells via endogenously expressed ACE2 receptors (21). We hypothesized that ACE2 overexpression could enhance Vero cell susceptibility to VSV-SARS2-Fluc and thereby improve assay sensitivity. To this end, we tested Vero-ACE2 cells, which stably overexpress human ACE2 as confirmed by flow cytometry (Fig. 2H and I), in the assay. While Vero and Vero-ACE2 cells naturally express relatively high levels of TMPRSS2 (Fig. 2J and K), we also generated a stable cell line overexpressing both ACE2 and TMPRSS2 to elucidate the effect of TMPRSS2 on assay performance. VSV-SARS2-Fluc infection induced higher luciferase expression in Vero-ACE2 cells than in Vero cells (Fig. 3A). Luciferase expression was not further enhanced by overexpression of TMPRSS2, and notably, VSV-SARS2-Fluc neutralization by the well-characterized neutralizing anti-SARS-CoV-2 spike monoclonal antibody mAb10914 was less apparent on Vero-ACE2/TMPRSS2 cells than on Vero-ACE2 cells (Fig. 3A). Since Vero-ACE2 cells provided for more sensitive detection of viral neutralization, these cells were selected as the cell substrate for assay development.

We also examined the effect of Vero-ACE2 cell seeding density on assay performance. Higher luciferase activity was detected when cell density was increased from 5,000 to 10,000 cells/well (96-well plate; Fig. 3B), but further increasing the cell density to 20,000 cells/well led to only a modest additional incremental increase in luciferase signal. Moreover, the higher cell density of 20,000 cells/well was associated with a less effective neutralization of luciferase signal when the virus was exposed to the neutralizing antibody mAb10914. We concluded that 10,000 cells/well was the optimal seeding density for detection of virus neutralization.

To demonstrate the detection of neutralizing antibodies in patient samples, we used serum samples confirmed as seronegative or seropositive by the commercial EUROIMMUN anti-SARS-CoV-2 ELISA (IgG), which detects anti-SARS-CoV-2 spike antibodies. Serum samples were incubated with VSV-SARS2-Fluc for 30 min at room temperature and then added to culture wells containing preplated Vero-ACE2 cells. All five of the seropositive samples substantially inhibited virus infection, resulting in suppression of luciferase activity (Fig. 3C). No reduction in luciferase activity was observed when the VSV-SARS2-Fluc virus was preincubated with seronegative samples, confirming that neutralizing antibodies were detected only in seropositive donor samples.

### Consistency of different VSV-SARS2-Fluc production lots.

**FIG 4:**
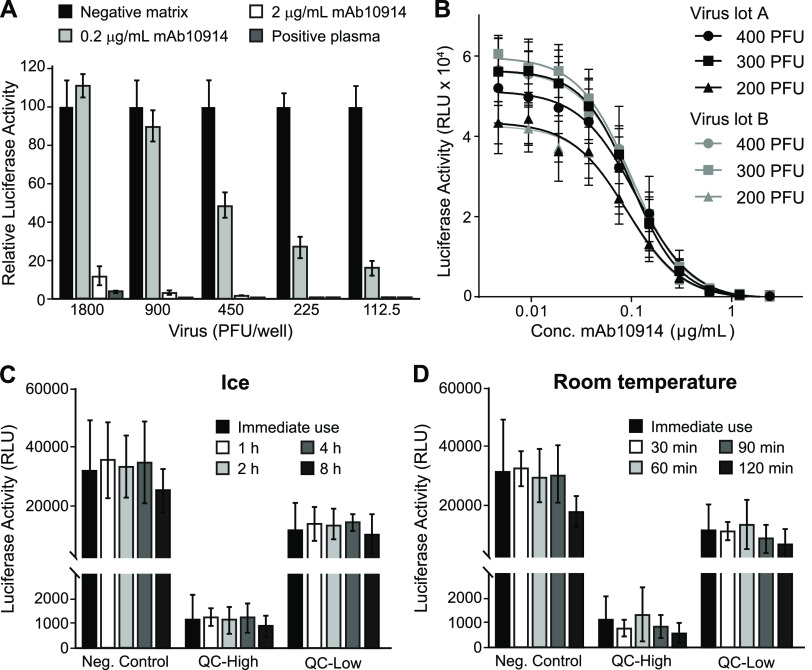
Assay performance of VSV-SARS2-Fluc. (A) Susceptibility of virus to antibody neutralization. The indicated amounts (PFU) of VSV-SARS2-Fluc were incubated with 2 or 0.2 μg/ml anti-SARS-CoV-2 spike monoclonal antibody mAb10914, a SARS-CoV-2-seropositive plasma sample at a 1:80 dilution, or pooled seronegative serum (negative matrix, 1:80). After 30 min, virus mixes were overlaid onto Vero-ACE2 cells, and luciferase activity was measured after an additional 24 h. Values represent the average (mean) luciferase activity relative to the negative matrix control ± standard deviation. (B) Consistency of virus lots. Various amounts (PFU) of two different lots (A and B) of VSV-SARS2-Fluc were incubated with the indicated concentrations of mAb10914. Luciferase activity was measured after an additional 24 h. Values represent the average (mean) RLU ± standard deviation. (C and D) Virus stability. Aliquots of VSV-SARS2-Fluc were removed from the freezer, thawed, and either used immediately for assay (Immediate use) or stored either at room temperature or on ice for the indicated time (h). For assay, 300 PFU of VSV-SARS2-Fluc was incubated with 0.154 (QC-High) or 0.031 (QC-Low) μg/ml of anti-SARS-CoV-2 spike monoclonal antibody mAb10922 in pooled seronegative sera or in pooled seronegative sera alone (Neg. Control). After 30 min, virus mixes were overlaid onto Vero-ACE2 cells, and luciferase activity was measured after an additional 24 h. Values represent the average (mean) RLU ± standard deviation.

To determine the optimal quantity of virus to add to each assay well, we tested the capacity of mAb10914 and seropositive plasma to neutralize increasing amounts of VSV-SARS2-Fluc. Highly neutralizing seropositive plasma and mAb10914 at a concentration of 2 μg/ml inhibited infectivity by at least 90%, independently of the amount of virus added to the well (Fig. 4A). In contrast, mAb10914 at a concentration of 0.2 μg/ml noticeably blocked infectivity in this assay only when less than 900 PFU of virus was added to each well. Based on this experiment, the optimal quantity of VSV-SARS2-Fluc virus to be added to each well to ensure sensitive detection of low levels of neutralizing antibodies is between 200 and 400 PFU. Consistency of virus lots was confirmed by comparing mAb10914 inhibition of two independent lots of VSV-SARS2-Fluc (produced at different times and representing subsequent virus passages). Luciferase activity over a range of concentrations of mAb10914 was nearly indistinguishable between the two different virus lots (Fig. 4B). Comparing the mAb10914 inhibition curve with 200, 300, and 400 PFU of virus per well, the linear range was slightly wider when 300 PFU/well of VSV-SARS2-Fluc was used. Therefore, we used 300 PFU for all future assay runs. We also tested the stability of the thawed VSV-SARS2-Fluc virus when stored on ice or at room temperature prior to being used in the assay. No significant reduction in virus infectivity or neutralization occurred following an 8-h incubation on ice (Fig. 4C). Likewise, the virus was stable for up to an hour at room temperature, with only a modest titer decrease noted after 2 h (Fig. 4D).

### Heat inactivation of serum samples is not necessary for assay compatibility.

**FIG 5:**
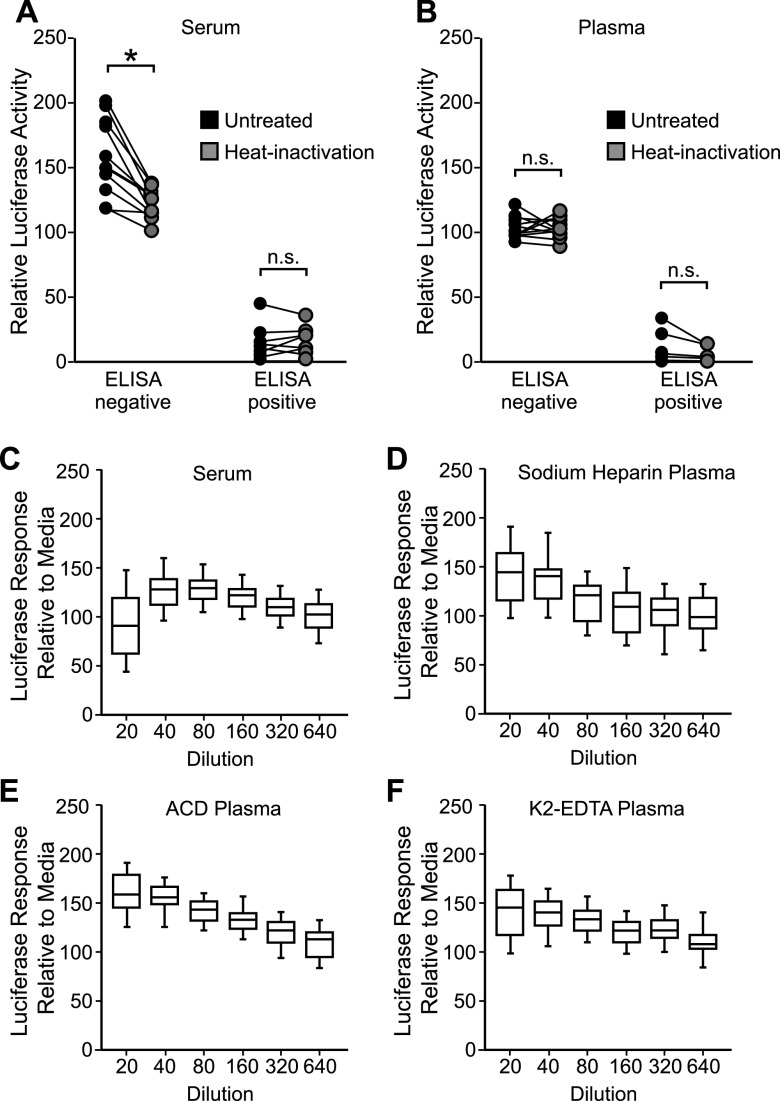
Effect of sample matrix on assay performance. (A and B) Effect of heat inactivation of sera or plasma. Matched serum (A) and sodium-heparin plasma (B) samples from 20 donors (11 seronegative and 9 seropositive for anti-SARS-CoV-2 antibodies by ELISA) were split and incubated either on ice or at 56°C for 30 min. Following incubation, plasma samples were clarified by centrifugation. Samples were then incubated at a 1:80 dilution with VSV-SARS2-Fluc. Pooled seronegative sera or plasma were used as assay controls. After 30 min, virus mixes were overlaid onto Vero-ACE2 cells, and after an additional 24 h, luciferase activity was measured. Values represent the average (mean) luciferase activity relative to the pooled seronegative matrix control for each sample. Statistical analysis was performed using a two-way repeated-measures (RM) analysis of variance (ANOVA) followed by a Bonferroni multiple-comparison test. n.s., not significant; * = <0.0001. (C to F) Characterization of matrix interference. Seronegative serum (C, *n* = 40), sodium-heparin plasma (D, *n* = 40), ACD plasma (E, *n* = 26), or K_2_-EDTA plasma (F, *n* = 49) samples were serially diluted as indicated and incubated with VSV-SARS2-Fluc. Virus mixed with medium only was used as a control. After 30 min, virus mixes were overlaid onto Vero-ACE2 cells, and after an additional 24 h, luciferase activity was measured. Values represent the average (mean) luciferase activity relative to the medium control ± standard deviation.

In cellular assays, heat inactivation of plasma and serum samples is often necessary to limit matrix interference that can affect cell or virus viability. To determine whether heat inactivation was required for IMMUNO-COV v2.0, 20 matched serum and plasma samples were thawed and aliquoted, with one aliquot kept on ice while the other aliquot was heat inactivated at 56°C for 30 min. Both aliquots were then tested in the assay. Overall, heat inactivation had little effect on neutralizing activity. All seronegative samples remained negative and all seropositive samples remained positive in the assay, regardless of whether the samples had been heat inactivated (Fig. 5A and B), though heat inactivation did reduce seronegative serum enhancement of virus infection. Importantly, heat-inactivated samples did not exhibit diminished virus-neutralizing capacity, suggesting that complement proteins do not enhance the neutralization of VSV-SARS2-Fluc in this assay format. For plasma samples, heat inactivation and subsequent clarification prevented thermal coagulation and sample loss during the assay, thereby improving assay performance. We therefore continued to use heat inactivation for all subsequent assays with plasma samples, while using non-heat-inactivated serum samples.

### Serum and plasma demonstrate low matrix interference.

In our original cell fusion-based IMMUNO-COV assay, we observed significant matrix interference at high concentrations of serum and plasma (21). To determine whether IMMUNO-COV v2.0, which provides a more direct measure of virus infection, is similarly hampered by matrix interference, we ran numerous seronegative samples in the assay at 2-fold serial dilutions ranging from 1:20 through 1:640. Minimal matrix interference was observed with serum, sodium-heparin plasma, acid citrate dextrose (ACD) plasma, and K_2_-EDTA plasma (Fig. 5C to F). In fact, higher concentrations of plasma appeared to have a stabilizing effect on the virus relative to cell culture medium alone and were associated with higher levels of luciferase activity at assay readout. Likewise, serum appeared to increase virus stability relative to medium alone, though some matrix interference was observed at the 1:20 dilution. Thus, the IMMUNO-COV v2.0 assay is compatible with testing at low sample dilutions, which may be of importance if higher detection sensitivities are desired.

### Quantification of neutralizing antibody titers using a standard curve.

**FIG 6:**
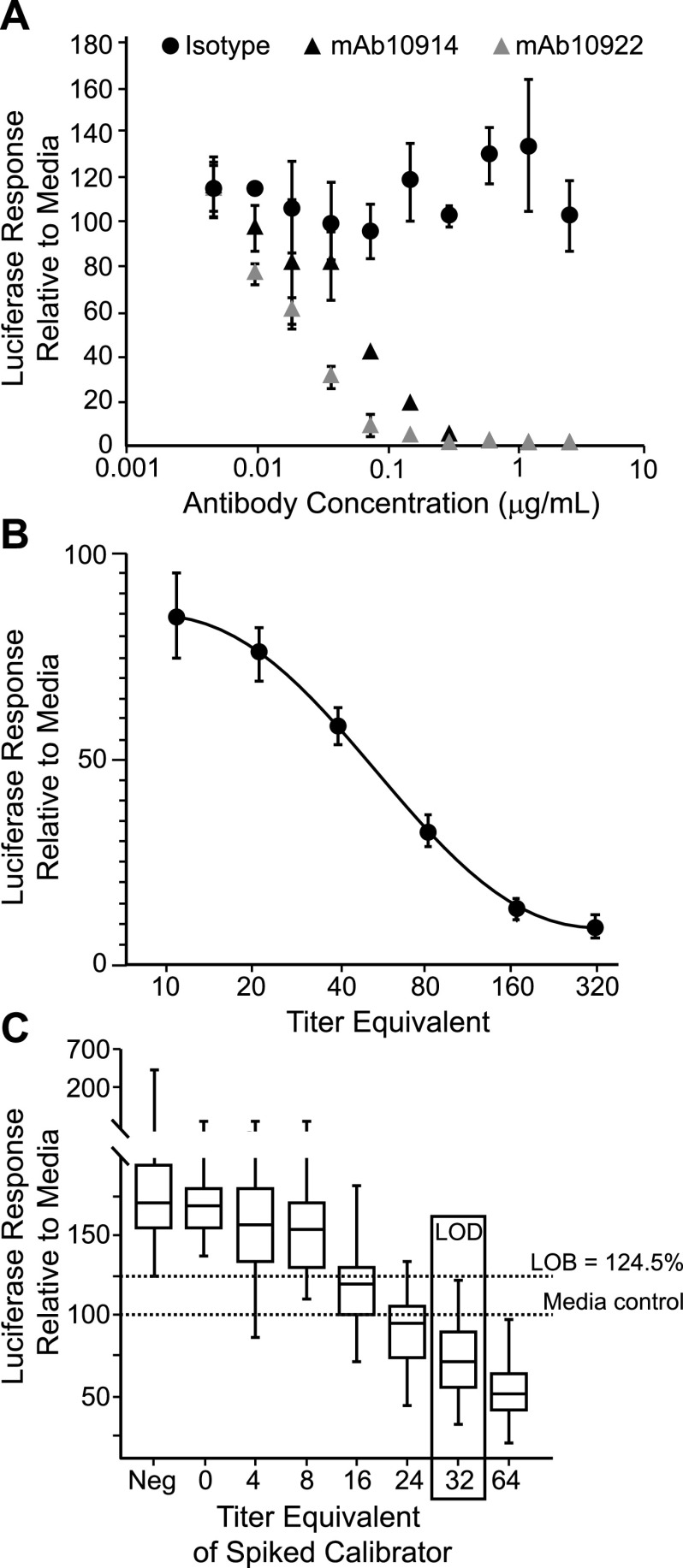
Establishment of a standard curve for titer calculations. (A) Antibody-specific neutralization of VSV-SARS2-Fluc. The indicated concentrations of anti-SARS-CoV-2 spike monoclonal antibody mAb10914 or mAb10922 or isotype control antibody were incubated with VSV-SARS2-Fluc. After 30 min, virus mixes were overlaid onto Vero-ACE2 cells, and luciferase activity was measured after an additional 24 h. Values represent the average (mean) luciferase activity relative to the medium control ± standard deviation. (B) Standard curve performance. VSV-SARS2-Fluc was incubated with 0.8, 0.4, 0.2, 0.1, 0.05, or 0.025 μg/ml (corresponding to the indicated equivalent VNTs) of mAb10914 or negative pooled sera alone. After 30 min, virus mixes were overlaid onto Vero-ACE2 cells, and luciferase activity was measured after an additional 24 h. Values represent average (mean) luciferase activity relative to the pooled negative serum control ± standard deviation from 242 unique assay runs. (C) Limit of detection. Five different seronegative serum samples (at a 1:80 dilution) were spiked with anti-SARS-CoV-2 spike monoclonal antibody mAb10914 at 0.01, 0.02, 0.04, 0.06, 0.08, and 0.1 μg/ml (corresponding to the indicated equivalent VNTs) and incubated with VSV-SARS2-Fluc. VSV-SARS2-Fluc incubated with unspiked serum samples (Neg) or medium alone were included as controls. After the 30-min incubation, virus mixes were overlaid onto Vero-ACE2 cells, and luciferase activity was measured after an additional 24 h. Box and whisker diagrams display the interquartile range in the box, with the center line representing the median for the data set and whiskers representing the lower 5% and upper 95% value. Values are based on a total of 12 different assay runs performed on three separate days by six analysts using two different virus lots.

To determine the titer of neutralizing antibodies in a test sample without the need for serial 2-fold sample dilutions, we developed an assay format in which just one or two dilutions of a test sample are read against a standard calibration curve included on every assay plate. For the development of a calibration standard and assay controls, we used two well-characterized neutralizing anti-spike monoclonal antibodies, mAb10914 and mAb10922. Both antibodies neutralized VSV-SARS2-Fluc in a dose-dependent manner (Fig. 6A), whereas no virus inhibition was observed using isotype antibody at any of the concentrations tested. Based on these findings, we established a six-point standard curve using 2-fold dilutions of mAb10914 in tissue culture medium at concentrations ranging from 0.8 μg/ml to 0.025 μg/ml (Fig. 6B). To quantify the viral neutralizing titers of test samples, each antibody concentration in the standard curve was converted to a virus-neutralizing titer (VNT) by multiplying the antibody concentration by 400. The correction factor of 400 was chosen as it produced VNT values that approximated PRNT50% values obtained for samples assayed at a 1:80 dilution (see below). The final standard curve range for the assay therefore gives a VNT readout of 10 to 320 for a sample assayed at a 1:80 dilution. In numerous tests (*n* = 242 assay runs), the 160, 80, 40, and 20 VNT standards fell within the linear range >99% of the time (Table 1). In most runs (87.6%), either the 320 or 10 VNT standard was also within the linear range. Thus, the standard curve effectively spanned the assay linear range. To quantitate antibody titers above 320 VNT, additional sample dilutions above 1:80 were employed in the assays described below.

**TABLE 1:** Assay linearity

Standard^*c*^	ST1	ST2	ST3	ST4	ST5	ST6
Nominal value^*a*^	320	160	80	40	20	10
Mean value	278.0	167.3	80.3	39.3	20.4	10.7
SD	23.7	21.4	8.7	3.1	1.7	1.3
%CV	8.5	12.8	10.8	8.2	8.1	12.0
%RE	−13.1	4.6	0.4	−1.9	1.9	7.3
% in range^*b*^	59.5	99.2	99.6	100.0	99.4	39.4
No. in range^*b*^	144	240	241	242	226	95

a Expected VNT value based on concentration of mAb10914 in each standard.

b Total *n* from all runs is 242.

c Abbreviations: SD, standard deviation; CV, coefficient of variation; RE, relative error.

### Under standard conditions, the assay LOD is 32 VNT.

To determine the limit of detection (LOD) of the assay, we first determined the assay limit of blank (LOB), representing the background signal from seronegative serum. To this end, we assayed seven known seronegative serum samples at a 1:80 dilution on 12 assay runs and calculated the luciferase signal as a percentage of the signal in medium-only controls. As observed previously (Fig. 5C), seronegative samples stabilized virus, and the LOB was a luciferase response of 124.5% compared to medium alone. Seronegative serum samples were subsequently spiked with low concentrations of standard mAb10914 (0.01, 0.02, 0.04, 0.06, 0.08, and 0.1 μg/ml, corresponding to VNTs of 4, 8, 16, 24, 32, and 40, respectively) and assayed side-by-side with unspiked samples (Fig. 6C). Based on a total of 60 values obtained for each spike level, the lowest concentration of mAb10914 at which ≥95% of the luciferase response values were below the LOB was 0.08 μg/ml. This concentration corresponded to a VNT of 32, which was accepted as the LOD for the assay.

### The assay exhibits high specificity and sensitivity.

To evaluate the sensitivity and specificity of IMMUNO-COV v2.0 when used to discriminate between positive and negative results, we performed blind testing of 176 serum samples that were categorized as either positive or negative for SARS-CoV-2-neutralizing antibodies based on the readouts from ELISA and gold standard PRNT. All samples that tested positive for SARS-CoV-2 spike binding antibodies by ELISA were subsequently analyzed by PRNT, with only those samples that were positive by PRNT considered positive for neutralizing antibodies. Samples that tested negative by ELISA but positive in the IMMUNO-COV v2.0 assay were also tested by PRNT to confirm the presence or absence of neutralizing antibodies. In these analyses, our assay demonstrated 100% specificity compared to both PRNT50% and PRNT80% results, as all PRNT-negative samples tested negative in IMMUNO-COV v2.0 (Table 2). Assay sensitivity was 93.7% relative to PRNT50% and 98.3% relative to PRNT80%. Moreover, 137 serum samples acquired prior to March 2020 (132 acquired from 2017 to 2019, 5 acquired in early 2020) from donors recovered from infection with endemic human coronavirus HKU1 (*n* = 35), NL63 (*n* = 32), OC43 (*n* = 35), or 229E (*n* = 35) were all negative for neutralizing antibodies when tested using the IMMUNO-COV v2.0 assay. Thus, the assay specifically detected neutralizing antibodies to SARS-CoV-2 and most likely does not cross-react to the four common human coronaviruses.

**TABLE 2:** Assay specificity and sensitivity

IMMUNO-COV	PRNT50%^*a*^		PRNT80%^*a*^	
	Positive	Negative	Positive	Negative
Positive	59	0	59	0
Negative	4	113	1	116
Total	63	113	60	116
	Sensitivity: 93.7%	Specificity: 100.0%	Sensitivity: 98.3%	Specificity: 100.0%

a All samples were assayed by EUROIMMUN IgG ELISA. Any samples collected from donors previously PCR positive for SARS-CoV-2 or positive for SARS-CoV-2 antibodies by IMMUNO-COV or EUROIMMUN IgG ELISA were also tested by PRNT assay, which was used as the gold standard to assign seropositive and seronegative samples based on either PRNT50% or PRNT80% titers.

**FIG 7:**
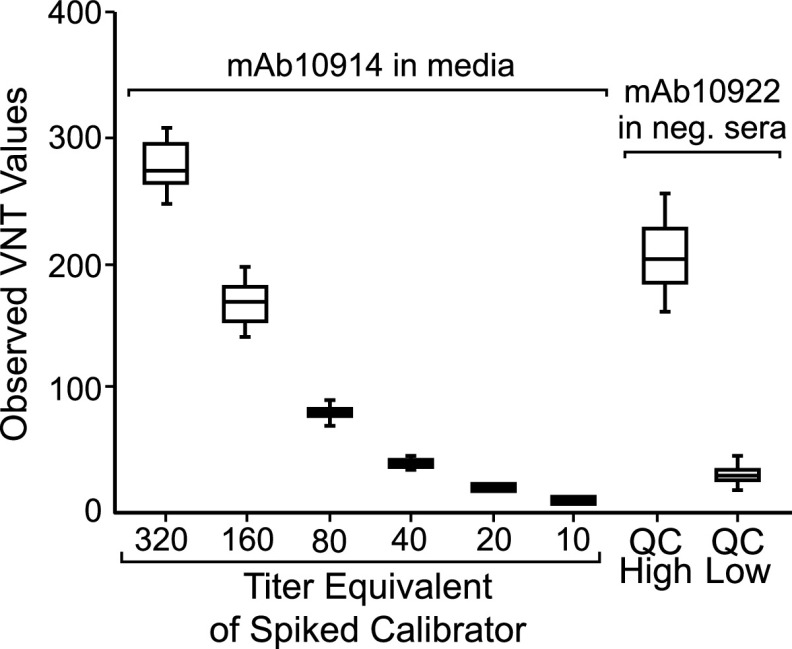
Interassay variability of standards and controls. Standards consisting of monoclonal anti-SARS-CoV-2 spike antibody mAb10914 at 0.8, 0.4, 0.2, 0.1, 0.05, and 0.025 μg/ml in media and QC High and QC Low controls consisting of 0.154 and 0.031 μg/ml antibody mAb10922, respectively, in pooled seronegative sera were incubated with VSV-SARS2-Fluc. Pooled seronegative serum alone was used as a negative control. After 30 min, virus mixes were overlaid onto Vero-ACE2 cells, and luciferase activity was read after an additional 24 h. A total of 207 assay runs were performed over 5 days, by five analysts, using two different virus lots. Box plot represents the 25th to 75th percentile of the data with the line representing the medium titer equivalent (VNT) value. Whiskers display the minimum and maximum values.

We also assessed assay variability. Each of the serum samples was assayed in a blind manner on five distinct runs performed by four different operators over a period of 5 days. Perfect consensus of positive and negative results between all five runs was observed for 174 (98.9%) of the samples, with just two positive samples of low titer testing negative in two out of five assay runs. Antibody titers of positive samples were consistent between operators and assay runs, with titers across five different runs exhibiting 27.9% coefficient of variation (CV) (*n* = 59), which compared favorably to a CV of 65.1% for the PRNT (*n* = 8 samples, two separate runs). Interassay precision was also evaluated based on the performance of the standard curve and assay controls. For this purpose, we included quality control (QC) High (0.154 μg/ml) and QC Low (0.031 μg/ml) controls consisting of mAb10922 spiked into negative pooled sera on each assay plate. From 207 assay runs, QC High and QC Low VNT readouts both demonstrated less than 30% interassay variability (Table 3 and Fig. 7). Intra-assay variability, which was assessed by running the same controls in 24 wells of the same plate, was below 20% for both controls (QC High = 8.6%, QC Low = 19.1%). Collectively, these data demonstrate that the IMMUNO-COV v2.0 assay has acceptably low levels of intra- and interassay variability.

**TABLE 3:** Intra- and interassay variability^*b*^

QC level	QC High			QC Low			Matrix blank
Predicted VNT^*a*^	160			32			0
Precision criterion	% response	VNT	Intra-assay %CV	% response	VNT	Intra-assay %CV	Intra-assay %CV
Mean value	2.8	208.6	13.3	37.7	29.8	21.4	9.8
SD	1	37.7		11.5	8.6		
%CV	37.8	18.1		30.6	28.8		

a Predicted VNT of QC samples based on concentration of mAb10922 spiked into matrix blank.

b Abbreviations: SD, standard deviation; CV, coefficient of variation; *n *= 207.

### Assay equivalence of serum and plasma samples.

While most of our assay validation studies were conducted using serum samples, we also performed matrix equivalency testing to confirm assay compatibility with different plasma matrices. To this end, we acquired matched serum, sodium heparin plasma, ACD plasma, and K_2_-EDTA plasma samples from 26 of the 176 subjects whose serum samples were used to evaluate assay specificity and sensitivity and tested the matched samples side-by-side in the assay. The consensus results and VNT antibody titers of positive samples from five assay runs were compared for each matrix. The average percent relative error for each matrix was within ±30% for all plasma matrices (+9.3%, −9.8%, and +24.6%, respectively, for sodium heparin plasma, ACD plasma, and K_2_-EDTA plasma). Although all three plasma matrices demonstrated equivalency in this experiment, in other experiments (data not shown) the sodium heparin plasma samples did not exhibit dilutional linearity. Thus, only ACD plasma and K_2_-EDTA plasma are currently considered acceptable matrices for clinical testing.

### IMMUNO-COV v2.0 VNT antibody titers correlate closely with PRNT50% titers.

**FIG 8:**
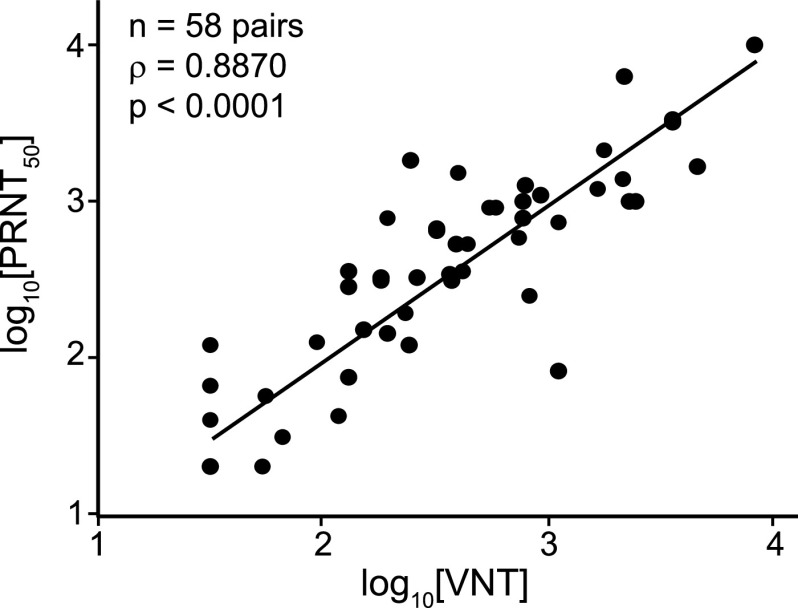
Correlation of virus-neutralizing units with PRNT50%. Fifty-eight SARS-CoV-2-seropositive serum samples were assayed using IMMUNO-COV v2.0 starting at a 1:80 dilution. Established controls, including a standard curve (0.8, 0.4, 0.2, 0.1, 0.05, and 0.025 μg/ml mAb10914 in medium), were included on each assay plate. The IMMUNO-COV v2.0 titer (VNT) was determined using the standard curve, where 1 VNT equals the concentration of mAb10914 multiplied by 400. All samples were subjected to PRNT using a clinical isolate of SARS-CoV-2. Statistical comparison of VNT to PRNT50% was performed using Spearman’s rank order correlation analysis as both data sets had a non-Gaussian distribution (*P* < 0.0001).

The BSL-3 PRNT with wild-type SARS-CoV-2 remains the gold standard for detection of neutralizing antibodies. Therefore, we compared the titers (VNT) measured using IMMUNO-COV v2.0 with those determined by PRNT. A strong correlation (Pearson’s *R* = 0.8870, *P* < 0.0001) was observed between VNTs and PRNT50% titers (Fig. 8). Therefore, neutralization of VSV-SARS2-Fluc in our assay closely mirrors the neutralization of SARS-CoV-2, and IMMUNO-COV v2.0 titers provide an accurate measure of an individual’s level of neutralizing antibodies. Moreover, VNTs can be quickly compared to PRNT50% titers using a conversion table (Table 4), which we generated based on our data obtained using the two different assays.

**TABLE 4:** VNT-to-PRNT50% conversion

VNT	PRNT50%
<32	<1:40
32 to 40	1:40
41 to 80	1:80
81 to 180	1:160
181 to 400	1:320
401 to 800	1:640
801 to 1,600	1:1,280
1,601 to 2,400	1:2,560
>2,400	>1:2,560

### Individuals with more severe disease symptoms tend to develop higher titers of neutralizing antibodies.

**FIG 9:**
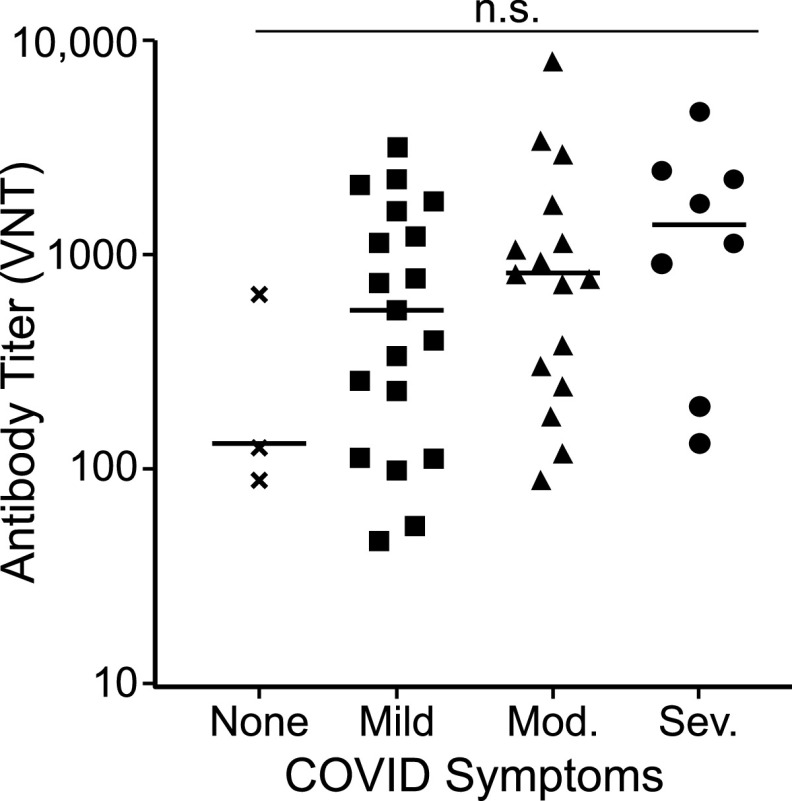
The strength of neutralizing antibody responses correlates with disease severity. As part of assay validation (Table 2), neutralizing antibody titers were determined for 46 donors who self-reported COVID-19 disease symptoms at least 2 weeks prior to sample donation. Disease symptoms were classified as severe (acute respiratory distress or pneumonia), moderate (shortness of breath), mild (fever, feverishness, cough, chills, myalgia, rhinorrhea, sore throat, nausea/vomiting, headache, abdominal pain, or diarrhea), or none (asymptomatic). The graph indicates the titer value (VNT) for each donor grouped based on disease symptoms. Bars represent the average (mean) titer for each group. Differences in antibody titers based on disease severity were not statistically significant (n.s.) by one-way ANOVA (*P* = 0.1904).

Increasing evidence indicates that disease severity influences the strength of the neutralizing antibody response (7, 8, 16, 28, 32). To examine whether individuals in our study with more severe disease developed higher titers of neutralizing antibodies, we correlated antibody titers with self-reported disease symptoms from 46 previously infected donors who had tested positive for SARS-CoV-2-neutralizing antibodies. Samples used for this analysis were collected within the time window of 2 weeks to 2 months after confirmation of COVID-19 diagnosis. A wide range of neutralizing antibody titers was observed among these donors with significant overlap between the disease severity groupings (Fig. 9). Mean neutralizing antibody titers increased with increasing disease severity, though differences were not statistically significant. Our data, therefore, support previous findings that strong neutralizing antibody responses are more likely in individuals who have recovered from severe disease, but wide variation in neutralizing titers occurs within all disease severity groupings.

### SARS-CoV-2-neutralizing antibody titers fall steadily after recovery from infection.

**FIG 10:**
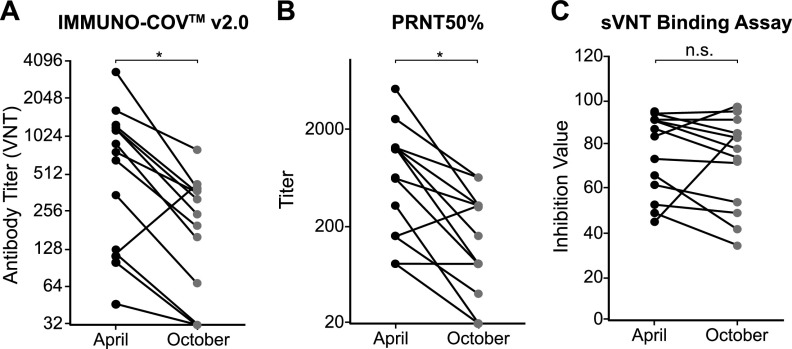
Durability of neutralizing antibody responses. (A to C) Samples were collected from donors in April and October 2020 (*n* = 13). Neutralizing antibody levels were measured using IMMUNO-COV v2.0 (A), the PRNT assay (B), or the c-PASS SARS-CoV-2 neutralization antibody detection kit (C), which is a binding assay that utilizes the SARS-CoV-2 spike RBD. The reductions in antibody titers were statistically significant (*) for IMMUNO-COV v2.0 and the PRNT assay but not significant (n.s.) for the sVNT binding assay (*P* = 0.0007, 0.0004, and 0.4669, respectively, from paired *t* test).

To provide long-term protection from COVID-19, neutralizing antibodies must persist at sufficiently high levels to block infection or mitigate pathogenesis. To examine the durability of SARS-CoV-2-neutralizing antibodies after recovery from natural infection, we determined the change in neutralizing antibody titers from 13 subjects between April and October 2020. In April, all 13 of these subjects had been diagnosed with COVID-19 within the previous 2 months and had measurable levels of SARS-CoV-2-neutralizing antibodies. Samples collected in April were stored at ≤−65°C and assayed side-by-side with new samples collected in October from the same subjects. A 2- to 5-fold drop in neutralizing antibody titers was observed in all but one subject (Fig. 10A and Table 5). The outlier showed a 3-fold increase, suggesting possible asymptomatic reexposure to the virus. In three subjects, the VNT from October dropped below the limit of detection in serum, though neutralizing antibodies could still be detected at very low levels in ACD plasma from two of these subjects. Together, these data indicate that SARS-CoV-2-neutralizing antibody titers fall quite rapidly over time following natural infection. Importantly, while the PRNT confirmed the substantial decrement in SARS-CoV-2-neutralizing antibody titers over 6 months (Fig. 10B), a similar trend was not observed using a “neutralization” assay that measures binding of the spike RBD to immobilized ACE2 receptor (Fig. 10C and Table 5). When samples were tested using this SARS-CoV-2-spike RBD binding assay, antibody levels in several subjects were similar in April and October. This finding highlights the importance of quantifying neutralizing antibodies by inhibition of live virus rather than relying on a surrogate receptor binding assay.

**TABLE 5:** Longevity of neutralizing antibodies

Donor	IMMUNO-COV v2.0 titer (VNT)			c-PASS value^*a*^		
	April^*b*^	October^*c*^	Relative titer	April^*b*^	October^*c*^	Relative value
1	1,652	784	0.47	94	95	1.01
2	1,187	320	0.27	84	96	1.14
3	47	<LOD	≤0.68	49	35	0.71
4	769	376	0.49	92	92	1.00
5	3,030	378	0.12	88	74	0.84
6	1,179	246	0.21	91	79	0.87
7	1,219	378	0.31	95	84	0.88
8	124	<LOD^*d*^	≤0.26	67	42	0.63
9	894	156	0.17	74	73	0.99
10	102	<LOD^*d*^	≤0.31	62	54	0.87
11	660	195	0.30	91	83	0.91
12	350	68	0.19	52	49	0.94
13	114	418	3.67	45	85	1.89

a Samples analyzed using the c-PASS SARS-CoV-2 surrogate virus neutralization test (sVNT) kit.

b April samples acquired from donors 2 to 8 weeks following COVID-19 symptoms or diagnosis.

c October samples acquired from same donors approximately 6 months after April samples acquired.

d Matched ACD plasma samples were also analyzed and exhibited low levels of neutralizing antibodies in some assay runs.

## DISCUSSION

With vaccine rollout ongoing and critical questions still unanswered regarding the durability of protective immune responses, the need for an accurate, scalable test that can quantitatively measure SARS-CoV-2-neutralizing antibodies remains a priority. Only a small subset of antibodies capable of binding to the spike glycoprotein have neutralizing activity and are most likely to afford protection against SARS-CoV-2 infection (16, 27, 29, 33). Commercially available monoclonal antibodies proven to be of benefit for the treatment of COVID-19 were selected based on their potent virus-neutralizing activity (34–38). Yet, most serological tests currently in use detect total spike binding antibodies but do not measure the capacity of these antibodies to neutralize virus infectivity. The traditional assay for detection and quantification of neutralizing antibodies, the PRNT, is low in throughput and for SARS-CoV-2 must be performed under high biocontainment (BSL-3), making it impractical for widespread use. Here, we describe the development and clinical validation of a novel assay, IMMUNO-COV v2.0, which is now available as a scalable laboratory-developed test for quantitatively measuring SARS-CoV-2-neutralizing antibody titers. Our data show that IMMUNO-COV v2.0 can be used for accurate tracking of neutralizing antibody titers over time in individuals following natural infection (Fig. 10) or vaccination. Such information will be needed to better define what constitutes a protective immune response and what is the durability of the protective immune response following natural infection or vaccination. Answers to these questions will be important to better inform vaccine dosing schedules and other public health initiatives aimed at controlling the pandemic.

The IMMUNO-COV v2.0 assay measures the concentration of antibodies in serum or plasma that can neutralize the infectivity of the VSV-SARS2-Fluc virus in Vero-ACE2 cells, as detected by a reduction in luciferase activity compared to cells that have been infected in the absence of neutralizing antibodies (Fig. 1). Importantly, results from IMMUNO-COV v2.0 correlate closely with PRNT50% titers determined using a clinical isolate of SARS-CoV-2 (Fig. 8), indicating that neutralization of VSV-SARS2-Fluc accurately mirrors SARS-CoV-2 neutralization. Other groups have likewise observed strong correlation between the readouts of virus neutralization assays using VSV and lentiviral pseudotypes displaying the SARS-CoV-2 spike glycoprotein and readouts of classical PRNT conducted under BSL-3 using clinical isolates of SARS-CoV-2 (18, 22, 39). Given the strong correlations between titers determined using IMMUNO-COV v2.0 and those determined using classical PRNT50% and PRNT80% assays, we generated a conversion table that facilitates the rapid conversion of IMMUNO-COV v2.0 titers to corresponding PRNT50% titers (Table 4). Moreover, the VNT scale for IMMUNO-COV v2.0 was designed to yield numerical values roughly equivalent to the PRNT50% titers obtained for a given sample.

The currently available spectrum of tests for determining titers of SARS-CoV-2-neutralizing antibodies is based on clinical isolates of SARS-CoV-2 (PRNT) (40–42), replicating surrogate viruses (typically VSV derived) (20–22), nonreplicating spike protein pseudotyped viruses (primarily using VSV or lentiviruses) (10, 14, 17–19), or entirely nonviral platforms (RBD-ACE2 binding assays) (43, 44). Binding assays using spike receptor binding domain (RBD) are attractive due to the speed at which results can be obtained. However, they measure only that subset of neutralizing antibodies capable of blocking the binding of the SARS-CoV-2 spike protein RBD to its immobilized ACE2 receptor. They do not functionally measure virus neutralization, and since only a portion of SARS-CoV-2-neutralizing antibodies binds to the RBD (27, 29), the relevance of these assays relative to those that directly measure the inhibition of virus infection remains an open question. In relation to this important question, we observed similar trends between IMMUNO-COV v2.0 and PRNT50% titers from samples acquired at different times following SARS-CoV-2 infection. In contrast, we observed a much less robust correlation between PRNT50% titers and the c-PASS SARS-CoV-2 surrogate virus neutralization test kit, which is a spike RBD binding assay (Fig. 10).

In addition to comparing our assay to the gold standard PRNT assay, we performed full clinical validation of IMMUNO-COV v2.0, which included evaluating the parameters of linearity, assay dynamic range, sensitivity, determination of the limit of blank (LOB) and limit of detection (LOD), dilutional linearity and parallelism, precision, clinical agreement, matrix equivalence, clinical specificity and sensitivity, and assay robustness. IMMUNO-COV v2.0 exhibited excellent clinical agreement with 100% assay specificity (Table 2). We also tested samples obtained predominately before 2019 from individuals recovered from infection with one of the four common human coronaviruses (HKU1, NL63, OC43, and 229E). All these samples tested negative for neutralizing antibodies, suggesting that IMMUNO-COV v2.0 is specific to SARS-CoV-2-neutralizing antibodies and most likely will not detect neutralizing antibodies directed against other human coronaviruses.

As has been reported by others (7, 8, 16, 28, 32), we observed that donors recovering from more severe COVID-19 disease generally developed higher-titer neutralizing antibody responses (Fig. 9). However, several individuals with only mild COVID-19 symptoms developed strong neutralizing antibody responses, and two individuals with severe disease developed relatively weak neutralizing antibody responses. Thus, SARS-CoV-2-neutralizing antibody titers cannot be accurately predicted based on the severity of the disease manifestations that an individual experiences, highlighting the importance of neutralizing antibody testing to determine anti-SARS-CoV-2 immune status. Irrespective of the initial magnitude of the neutralizing antibody response, repeat IMMUNO-COV v2.0 testing demonstrated a 2- to 5-fold decline in SARS-CoV-2-neutralizing antibody titers over 6 months (Fig. 10). This finding is in keeping with those of other investigators (7–11) and highlights the importance of tracking neutralizing antibodies over time. It should be noted that some other studies suggest that SARS-CoV-2-neutralizing antibody titers are relatively stable (45, 46). More research is needed to better understand the durability of neutralizing antibody responses to SARS-CoV-2 and their relationship to cell-mediated responses. Further investigation is also needed to determine whether vaccination provides immunity against SARS-CoV-2 viral variants, and we are conducting ongoing studies to confirm that IMMUNO-COV v2.0 can detect immunity against SARS-CoV-2 variants.

It is not currently known what minimum titer of SARS-CoV-2-neutralizing antibodies is necessary to ensure protection against future infection. Likely, there will be considerable variation between individuals because of the multiple additional factors impacting susceptibility to infection, including age, sex, race, ethnicity, and various comorbid conditions. Nevertheless, it is widely accepted that higher levels of neutralizing antibodies afford a higher degree of protection from future infection. Large, coordinated studies following SARS-CoV-2-neutralizing antibody titers in various cohorts of vaccinated and previously infected individuals will be needed to understand immune correlates of protection, the durability of the protective response, and the appropriate frequency for administration of booster doses of the approved SARS-CoV-2 vaccines. With the advent of IMMUNO-COV v2.0, a fully validated, high-throughput laboratory-developed test that accurately and robustly determines neutralizing antibody titers, we can now move forward with these much-needed population studies. We have generated and cryopreserved sufficient VSV-SARS2-Fluc virus to perform over 5 million assays, and the assay is accurate and reproducible even between different virus lots (Fig. 4). Moreover, during validation testing, the IMMUNO-COV v2.0 assay exhibited favorable precision compared to the PRNT, with acceptable levels of intra- and interassay variability (Table 3) and low run-to-run variability in quantitative VNT readouts. Therefore, we believe that IMMUNO-COV v2.0 will provide a useful and lasting standardized assay that can be used to normalize and harmonize neutralizing antibody titers for consistent monitoring of neutralizing antibody levels over time and in large study populations.

## MATERIALS AND METHODS

### Cells.

African green monkey Vero cells (ATCC CCL-81), Vero-αHis (47), and baby hamster kidney BHK-21 cells (ATCC CCL-10) were maintained in high-glucose Dulbecco minimal essential medium (DMEM) supplemented with 5% fetal bovine serum (FBS) and 1× penicillin-streptomycin (complete medium) at 37°C and 5% CO_2_. Vero-ACE2-Puro (Vero-ACE2) cells were generated by transducing Vero cells with lentiviral vector LV-SFFV-ACE2-Puro, encoding the human ACE2 cDNA (GenBank accession no. BC039902) under the control of the spleen focus-forming virus (SFFV) promoter and linked to the puromycin resistance gene via a P2A cleavage peptide. Vero-ACE2-Puro/TMPRSS2-Puro (Vero-ACE2/TMPRSS2) cells were generated by transducing Vero-ACE2-Puro cells with lentiviral vector SFFV-TMPRSS2-Puro encoding human TMPRSS2 cDNA (GenBank accession no. BC051839) under the control of the SFFV promoter and linked to the puromycin resistance gene via a P2A cleavage peptide. Vectors used for stable-cell generation were verified by whole -plasmid sequencing performed by MGH CCIB DNA Core (Cambridge, MA). Transduced cells were selected using 10 μg/ml puromycin. Following selection, Vero-ACE2 cells were maintained in complete medium supplemented with 5 μg/ml puromycin. Puromycin was excluded when cells were seeded for assays.

### Generation of VSV-SARS2-Fluc.

Full-length Luc2 (Fluc) was PCR amplified from pLV-SFFV-Luc2-P2A-Puro (Imanis catalog no. DNA1034) with a 5′ NheI and 3′ AscI restriction site. To generate the viral genome, the amplified PCR product was cloned into pVSV-SARS-CoV-2-S-Δ19CT (21) between the SΔ19CT and L genes (Fig. 2A) using the NheI and AscI restriction sites. Plasmid was sequence verified and used for infectious virus rescue on BHK-21 cells as previously described (48). VSV-G was cotransfected into the BHK-21 cells to facilitate rescue but was not present in subsequent passages of the virus. For initial amplification, the virus was propagated in Vero-αHis cells by inoculating 80% confluent monolayers in 10-cm plates with 1 ml of virus. Virus was harvested 48 h after inoculation, aliquoted, and stored at ≤−65°C until use. For further amplifications and generation of large-scale stocks, the virus was propagated in Vero-ACE2 cells by inoculating 90% confluent monolayers at a multiplicity of infection (MOI) of 0.02 or 0.03 PFU per cell. Virus was harvested after 48 h, aliquoted, and stored at ≤−65°C until use. Aliquots were used to determine viral titers by plaque assay on Vero-αHis cells.

### Replication curves.

Vero-ACE2 or BHK-21 monolayers in 10-cm plates were inoculated in duplicate with Opti-MEM alone (mock), VSV-Fluc (MOI = 0.01), or VSV-SARS2-Fluc (MOI = 0.01). After 2 h at 37°C and 5% CO_2_, complete medium was added to a total volume of 6 ml/plate. At 2, 12, 24, 36, and 48 h, 0.25-ml aliquots of culture supernatant were removed from plates and replaced with 0.25 ml of fresh medium. Aliquots were stored at ≤−65°C immediately after collection until the time of titer determination. To determine viral titers, aliquots were thawed and assayed by plaque assay on Vero-αHis cells. Throughout the infection time course, cell photos were taken from the 10-cm plates at a ×100 magnification using an inverted microscope.

### Reagents.

D-luciferin potassium salt (Gold Biotechnology catalog no. LUCK-1G) was diluted in Dulbecco’s phosphate-buffered saline (DPBS) to generate 15-mg/ml stocks. For initial studies, 20 μl/well of stocks was used for assays. For later studies (starting with validation studies), stocks were diluted 1:10 in DPBS and 50 μl/well was used for assays. mAb10914 and mAb10922 are human anti-SARS-CoV-2 spike-neutralizing monoclonal antibodies. mAb10914 was prepared and scaled up using methods previously described by Regeneron Pharmaceuticals, Inc. (35), and mAb10922 was purchased from GenScript (catalog no. U314YFG090_1).

### Luciferase assay time course.

Vero-ACE2 or BHK-21 cell monolayers in 96-well black-walled plates with clear bottoms were infected with VSV-Fluc or VSV-SARS2-Fluc at a multiplicity of infection of 0.03 PFU per cell. Medium-only wells were used as mock controls. For each condition, 24 wells were prepared to facilitate 8 time points done in triplicate. At 2, 12, 16, 20, 24, 28, 36, and 48 h after inoculation, D-luciferin was added to one set of triplicate wells and bioluminescence was immediately measured using a Tecan Infinite II instrument (100-ms integration, 100-ms settling time per well).

### Collection of plasma and serum samples.

A clinical protocol to collect blood samples for assay validation was reviewed and approved by Western IRB on 1 April 2020 (study ID: VYR-COV-001). Samples were obtained with informed consent, and the protocol was conducted under International Conference on Harmonization-Good Clinical Practice (ICH-GCP) and all applicable sections of the Code of Federal Regulations. Serum and plasma samples were collected in April 2020 from patients who had previously tested positive for SARS-CoV-2 infection by a PCR test, patients who had known exposure to individuals infected with SARS-CoV-2 and symptoms of COVID-19, and a cohort of patients with no known exposure to or symptoms of COVID-19 and presumed to be seronegative. Clinical information was self-reported. A total of 150 adult volunteers were enrolled and provided blood samples at BioTrial in Newark, NJ, and Olmsted Medical Center in Rochester, MN, in April 2020. A subset of 26 participants returned and volunteered a second blood sample 6 months later in October 2020.

Geisinger provided 137 frozen serum samples comprising the endemic human coronavirus panel. These samples were collected from subjects who had tested positive for the presence of coronavirus HKU1, coronavirus NL63, coronavirus OC43, or coronavirus 229E using the Geisinger respiratory pathogen panel PCR test (Geisinger Medical Labs) on average 282.5 days before the collection date (median, 129.3 days; range, 1,171.3 to 29.1 days).

### IMMUNO-COV v2.0 neutralization assays.

Except where noted during initial optimization experiments, Vero-ACE2 cells were seeded at 1 × 10^4^ cells/well in 96-well black-walled plates with clear bottoms 16 to 24 h before being used for neutralization assays. On the day of assay, test samples and controls were prepared and mixed with VSV-SARS2-Fluc in U-bottom suspension cell culture plates to a final volume of 240 μl/well. Any indicated antibody concentrations or sample dilutions represent the antibody concentration or sample dilution following mixing with virus. Except when noted otherwise, serum samples were thawed and used for assay without additional processing, while plasma samples were prepared by heat inactivation for 30 min at 56°C, followed by clarification at 12,000 × g for 5 min and transfer of the liquid supernatant to fresh tubes. During initial optimization experiments, various concentrations of virus were tested, but for all subsequent assays, virus was used at 300 PFU/well (300 PFU/100 μl in U-well mixtures). Virus, test samples, and controls were all diluted as appropriate in Opti-MEM to generate final concentrations. For each plate, a standard curve consisting of 0.8, 0.4, 0.2, 0.1, 0.05, and 0.025 μg/ml mAb10914 in Opti-MEM, and controls NC (pooled negative matrix at 1:80), QC High (0.154 μg/ml mAb1022 in pooled negative matrix at 1:80), and QC Low (0.031 μg/ml mAb10922 in pooled negative matrix at 1:80) were included. Virus mixes in U-well plates were incubated at room temperature for 30 to 45 min, and then 100 μl of mixes was overlaid onto the Vero-ACE2 monolayers in duplicate. Plates were returned to a 37°C and 5% CO_2_ incubator for 24 to 28 h. D-luciferin was then manually added to wells using a multichannel pipette, and luminescence was read immediately (30 to 90 s) after D-luciferin addition using a Tecan M Plex or Tecan Lume instrument (100-ms integration, 100-ms settling time per well).

### Determination of virus titers.

Virus-neutralizing titers (VNTs) were determined based on a calibration curve. The calibration curve was run on each plate and consisted of mAb10914 spiked into pooled SARS-CoV-2-seronegative sera at 0.8, 0.4, 0.2, 0.1, 0.05, and 0.025 μg/ml. From the calibration curve, the equivalent concentration of neutralizing antibody for a given luciferase signal was determined. To convert to VNT, the antibody equivalent concentration was multiplied by 400, a correction factor chosen to yield VNT values similar to PRNT50% values.

### Determinant of limit of blank (LOB) and limit of detection (LOD).

Seven known seronegative samples were analyzed at a 1:80 dilution on 12 different assay runs, performed on three consecutive days, by six different analysts, using two separate virus lots. Luciferase signal relative to a medium control was determined for each sample. The data sets were nonnormal by Anderson-Darling and Shapiro-Wilk tests, so the LOB was established using a nonparametric model with the 5th percentile value of relative luciferase response obtained for each data set. From this analysis, the LOB was a response level of 124.5%. To determine the LOD, five seronegative samples (at a 1:80 dilution) were spiked with low levels of calibrator material (mAb10914) at 0.01, 0.02, 0.04, 0.06, 0.08, or 0.1 μg/ml and assayed on 12 different assay runs, performed on three consecutive days, by six different analysts, using two separate virus lots. Every run also included unspiked negative samples and medium control. Data sets were evaluated for the titer that resulted in a response level below the corresponding LOB for each of the dilutions. From these analyses, the LOD was determined to be 32 VNT.

### Blind testing of samples.

Serum and plasma samples were randomized by independent operators prior to being given to analysts for testing. Samples were assayed in batches, with an unknown number of positive and negative samples in each batch. All samples were assayed at 1:80, 1:160, 1:320, 1:640, 1:1,280, and 1:2,560 dilutions. For specificity and sensitivity studies, each sample was tested in a blind manner by four different analysts, on at least three different days, in a total of five separate assay runs, using two different virus lots. For comparison studies, samples were tested using the EUROIMMUN anti-SARS-CoV-2 ELISA (IgG) according to the manufacturer’s directions.

### Assay variability assessment.

QC High (0.154 μg/ml), QC Low (0.031 μg/ml), and matrix blank (0 μg/ml) controls consisting of mAb10922 diluted in pooled negative serum (at 1:80) were used along with the standard curve to assess assay variability. For interassay variability studies, controls were tested in duplicate on a total of 207 assay runs performed by five different analysts across a span of 5 days using two different lots of virus. For intra-assay variability studies, each control was assayed in 24 wells in the same assay run performed by the same analyst.

### Matrix equivalency assessment.

Matched serum, sodium heparin plasma, ACD plasma, and K_2_-EDTA plasma samples were obtained (see “Collection of plasma and serum samples”). Samples were assayed in a blind manner as described for blind testing of samples, using appropriate pooled negative matrix controls.

### PRNT.

Serum samples were heat inactivated for 30 min at 56°C and serially 2-fold diluted in Dulbecco’s minimal essential medium supplemented with 2% heat-inactivated fetal bovine serum. SARS-CoV-2 (USA-WA1/2020) (49) was diluted to approximately 200 PFU/ml and mixed with an equal volume of diluted serum (final dilutions of serum with virus were 1:20, 1:40, 1:80, 1:160, 1:320, 1:640, 1:1,280, 1:2,560, 1:5,120, 1:10,240, 1:20,480, and 1:40,960). Virus mixed with an equal volume of medium alone was used as a control. After 1 h of incubation at 37°C, 250 μl of virus-serum or virus-medium mixes was used to inoculate Vero-E6 monolayers in 6-well plates. Absorption proceeded for 1 h at 37°C with occasional rocking, before monolayers were overlaid with 4 ml of 1.6% low-melting-point agarose in minimal essential medium supplemented with 4% fetal bovine serum and antibiotics. Plates were incubated at 37°C for 2 days when plaques appeared and then fixed with 10% formaldehyde and stained with 2 ml of 0.05% neutral red, followed by incubation for 6 h at 37°C. Plaques were counted, and the PRNT50% and PRNT80% titers were determined as the lowest dilution at which the number of plaques was reduced by 50% or 80%, respectively, compared to the virus-medium control. Plaque counts greater than 30 were too numerous to count and were considered equivalent to the virus-medium control.

### sVNT binding assay.

Serum samples were tested using the SARS-CoV-2 surrogate virus neutralization test (sVNT) kit (GenScript catalog no. L00847) according to the manufacturer’s directions.

### Flow cytometry.

Vero-αHis (Vero) or Vero-ACE2 cells were dislodged using Versene, counted, and transferred to microcentrifuge tubes (5 × 10^5^ cells/tube were used for ACE2 staining and 1.5 × 10^6^ cells/tube were used for TMPRSS2 staining). For ACE2 staining, cells were pelleted and resuspended in 100 μl fluorescence-activated cell sorting (FACS) buffer (2% FBS in DPBS) containing 0.2 μg goat anti-human ACE2 (R&D Systems catalog no. AF933). After 30 min on ice, cells were rinsed with 1 ml FACS buffer and resuspended in 100 μl FACS buffer containing 5 μl donkey anti-goat IgG-phycoerythrin (PE) secondary antibody. After 30 min on ice, cells were rinsed with 1 ml FACS buffer and fixed with 1% paraformaldehyde for 15 min on ice. Cells were washed twice with FACS buffer, resuspended in 500 μl FACS buffer, and analyzed on a Cytoflex flow cytometer (Beckman Coulter). For TMPRSS2 staining, cells were resuspended in 1 ml ice-cold 70% ethanol in DPBS and incubated on ice for 10 min. Cells were centrifuged, washed once with 1 ml FACS buffer, and resuspended in 100 μl of a 0.5% saponin solution containing 4 μg rabbit anti-TMPRSS2 (Invitrogen catalog no. PA5-14264). After 30 min on ice, samples were washed twice with 1 ml FACS buffer and resuspended in 100 μl of a 0.5% saponin solution containing 2 μl goat anti-rabbit IgG-AF647 secondary antibody. After 30 min on ice, cells were washed twice with FACS buffer and fixed with 1% paraformaldehyde for 15 min on ice. Cells were washed twice with FACS buffer, resuspended in 500 μl FACS buffer, and analyzed on a Cytoflex flow cytometer (Beckman Coulter). For both ACE2 and TMPRSS2 staining, positive staining was compared against a control sample stained with secondary antibody only.

### Immunoblot assay.

Viruses were concentrated by high-speed centrifugation, and 5 × 10^5^ PFU (VSV-SARS2-Fluc) or 5 × 10^5^ 50% tissue culture infective dose (TCID_50_) units (VSV-GFP) were diluted in LDS sample buffer (Invitrogen catalog no. B0007) and reducing agent (Invitrogen catalog no. B0009) according to the manufacturer’s directions. Cell lysates from HEK-293T cells stably expressing SARS-CoV-2 spike protein were also prepared as controls. All samples were incubated at 70°C for 10 min, and 40 μl of each sample was run in duplicate on 4 to 12% Bis-Tris gels (Invitrogen catalog no. NW04125Box) along with Precision Plus protein dual color standard (Bio-Rad catalog no. 161-0374). Proteins were transferred to nitrocellulose membranes using a Power Blotter XL. Membranes were blocked in 5% nonfat dry milk in TBST, washed three times with TBST, and incubated for 1 h at room temperature with primary antibody mouse anti-SARS-CoV-2 spike (1:1,000; GeneTex catalog no. GTX632604) or mouse monoclonal anti-VSV-G clone 8G5F11 (1:10,000; Absolute Antibody catalog no. Ab01401-2.3). Membranes were washed three times with TBST and incubated for 1 h at room temperature with secondary antibody goat anti-mouse IgG-horseradish peroxidase (HRP) (Prometheus catalog no. 20-304) at 1:20,000. Membranes were washed three times with TBST, and protein bands were developed for 2 min at room temperature using ProSignal Dura ECL reagent (Prometheus catalog no. 20-301). Protein bands were imaged using a Bio-Rad ChemiDoc imaging system.

### Statistical analyses.

Descriptive statistics, comparisons, and regression analyses were performed in Graph Pad Prism, v9.0.0 (San Diego, CA). Tests for normality of variance were conducted, and whenever possible, parametric comparisons were used. For nonnormal data sets, nonparametric approaches were used. A four-parameter nonlinear regression was used for the calibration curve of the virus-neutralizing titer within the assay. For correlation analyses, Spearman’s correlation analysis was conducted.
